# A Randomized, Placebo Controlled Pilot Trial of Botulinum Toxin for Paratonic Rigidity in People with Advanced Cognitive Impairment

**DOI:** 10.1371/journal.pone.0114733

**Published:** 2014-12-23

**Authors:** Galit Kleiner-Fisman, Edwin Khoo, Nikohl Moncrieffe, Triina Forbell, Pearl Gryfe, David Fisman

**Affiliations:** 1 Jeff and Diane Ross Movement Disorders Clinic, Assistive Technology Clinic, Toronto, Canada; 2 Baycrest Center for Geriatric Health, University of Toronto, Toronto, Canada; 3 Department of Medicine, Division of Neurology, University of Toronto, Toronto, Canada; 4 Dalla Lana School of Public Health, University of Toronto, Toronto, Canada; University of Glasgow, United Kingdom

## Abstract

**Objective:**

Evaluate safety and efficacy of Incobotulinumtoxin A in elderly patients with dementia and paratonia.

**Setting:**

University-affiliated hospital, spasticity management Clinic.

**Participants:**

Ten subjects were enrolled. Inclusion criteria: 1) severe cognitive impairment 2) diagnosis of Alzheimer’s disease, vascular dementia, or frontotemporal dementia, and 3) score >3 on the paratonic assessment instrument, with posture in an arm(s) interfering with provision of care. Exclusion criteria: 1) alternate etiologies for increased tone and 2) injection with botulinum toxin within the 6 months preceding the study.

**Design:**

Single center, randomized, double blind, placebo-controlled, crossover trial with two treatment cycles of 16 weeks. Assessments occurred at 2, 6, 12 and16 weeks following injections. Subjects received up to 300 U of Incobotulinumtoxin A in arm(s).

**Primary and Secondary Outcome Measures:**

Primary outcome measure was the modified caregiver burden scale (mCBS); exploratory secondary outcome measures were also performed. Analysis of variance and mixed modeling techniques were used to evaluate treatment effects.

**Results:**

Incobotulinumtoxin A treatment produced significant improvement in mCBS total score −1.11 (–2.04 to −0.18) (Treatment effect and 95% CI), dressing sub-score −0.36 (–0.59 to 0.12), and cleaning under the left and right armpits sub-score −0.5 (–0.96 to −0.04), −0.41 (–0.79 to −0.04) respectively. PROM in the left and right elbow increased by 27.67 degrees (13.32–42.02) and 22.07 degrees (9.76–34.39) respectively. PROM in the left and right shoulder increased by 11.92 degrees (5.46–18.38) and 8.58 degrees (3.73–13.43) respectively. No significant treatment effect was found for GAS, VAS and PAINAD scales or change in time to perform care. No adverse drug reactions occurred.

**Conclusions:**

Administration of Incobotulinumtoxin A in elderly people with advanced dementia and paratonia may be an efficacious and safe treatment to increase range of motion and reduce functional burden. Further studies are needed to confirm results.

**Trial Registration:**

ClinicalTrials.Gov NCT02212119

## Introduction

It is frequently assumed that dementia is a disorder only of cognitive impairment; however, dementia is often accompanied by significant motor disability due in part to “paratonia”, a form of increased muscle tone. First observed by Dupre in 1910 [1], it was characterized as “an inability to relax muscles in the setting of cognitive impairment”. Paratonia has been estimated to be present in 5% of those with mild cognitive impairments and 100% in those with advanced dementia [2]–[5]. Postulated to originate in the central nervous system, paratonia exerts its effects by increasing muscle resistance reflexively when a limb is moved passively (though can fluctuate in severity depending on level of relaxation of the person). In advanced dementia, paratonia may also exist at rest with constant muscle contraction leading to fixed postures (contractures). Some of the consequences of paratonia and fixed postures include difficulties in washing, dressing, feeding, and providing general care, to a fully dependent person increasing caregiver burden. Fixed postures may lead to skin breakdown, infection, and pain upon movement, thereby reducing comfort and quality of life.

It is commonly accepted that once contractures have developed, medical treatments are futile. However, contractures may be avoided or delayed if paratonia is recognized and treated. Botulinum toxin type A (Xeomin, Botox, Dysport), is an exotoxin produced by the bacterium *Clostridium botulinum*. It can be used as a therapeutic intervention to selectively weaken skeletal muscle in a dose-related manner by impairing the release of acetylcholine, a neurotransmitter, at the neuromuscular junction [6]. Botulinum toxin is used widely to address other hypertonic states such as dystonia [7]–[10] and has been proven safe and effective for the treatment of post-stroke spasticity [11], [12]. In stroke patients with spasticity, the administration of Botulinum toxin has been shown to significantly improve the capacity to independently perform activities of daily living (ADLs) [11]. In those with severe disability requiring full care as the result of a stroke, administration of Botulinum toxin has been shown to reduce caregiver burden [12]. Injections are effective for approximately 3 months with peak effect occurring after ∼4–6 weeks. Unlike orally administered tone-reducing agents that may have substantial systemic consequences, side effects of Botulinum toxin are minimal as it only has local effects.

Given the safety of its use and proven effect on tone reduction in spasticity, we postulated that administration of Incobotulinum toxin A in cognitively impaired, dependent individuals with dementia exhibiting paratonic rigidity in the upper limbs would increase range of motion such that ADLs could be facilitated and discomfort and consequences of limb immobility could be delayed or prevented.

## Methods

### Subjects

The protocol for this trial and supporting CONSORT checklist are available as supporting information; see S1 Checklist and S1 Protocol.

Subjects were eligible for the study if 1) they had severe cognitive impairment defined as complete dependency in all ADLs; 2) diagnosed with of Alzheimer’s disease, vascular dementia, frontotemporal dementia (FTD), or mixed dementia not otherwise specified (NOS), and 3) scored >3 on the paratonic assessment instrument (PAI) [2] with consequent paratonic rigidity in one or both arm(s) interfering in the provision of care. Exclusion criteria included alternate etiologies for increased tone such as Parkinsonism, dystonia, territorial strokes or other focal neurological deficits, fixed contractures of the affected limb (assessed clinically as no mobility on passive range of motion), or injection with Botulinum toxin in the preceding 6 months. All participants were residents of a single long-term care facility. The Ethics Review Board at Baycrest Health Sciences approved the study. The study was approved on December 20^th^, 2010 with the first patient recruited April 11^th^, 2011 and the final patient recruited July 12^th^, 2012. The last follow-up was February 27^th^, 2013. All subjects had written informed consent provided by their power of attorney (POA).

Prior to enrollment of the first subject, the study was registered with Health Canada, the regulatory agency overseeing clinical trials in Canada where the study was performed. Technically, the trial didn’t have to be registered with clinicaltrial.gov as our trial did not meet criteria for “applicable drug clinical trial” subject to section 505 of the FDC Act or section 351 of the PHS Act. The drug was manufactured outside of the US and the trial was not being conducted under an investigational new drug (IND) designation [13].

However, given that registration of all clinical trials is now required for their acceptability for publication, the trial was retrospectively registered with clinicaltrials.gov. The authors confirm that all ongoing and related trials for this drug/intervention are registered.

### Study Design

**Figure 1:**
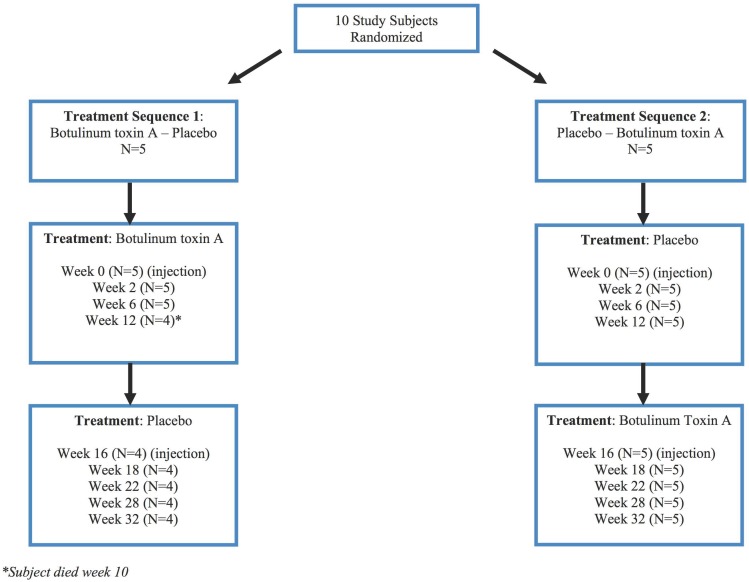
Study flow diagram.

The study was a single center, double blind, placebo-controlled, crossover trial with two treatment cycles of 16 weeks each (**Fig. 1**). The participants study involvement was 32 weeks. Following baseline assessments and injections at 0 and 16 weeks (either placebo-active drug or active drug-placebo sequence with sequence allocation randomly determined), repeated assessments took place at 2, 6,12 and 16 weeks following baseline assessment and injection. Though most Botulinum toxin studies use 12 weeks as the accepted time period for full washout, to reduce the possibility of carryover effect in those subjects randomized to receive Incobotulinumtoxin A first before placebo, we increased the study period to 16 weeks to ensure full washout. As this was a crossover design, each subject received both active drug and placebo. A pharmacist that had no involvement with patient care was responsible for preparation of the Botulinum toxin and placebo syringes and for their allocation to the treating physician based on the randomization sequence generated by a computer random number seed. Study investigators, treating physicians, patients and caregivers were all blinded to treatment allocation.

A single treating physician determined muscles chosen for injection and provided both placebo and active drug injections based on characteristics and magnitude of the posture causing disability (i.e. if elbow flexion was present biceps and brachioradialis muscles were injected, if shoulder abduction was present pectoralis muscle was injected, etc). Subjects were randomly assigned to receive up to a total of 300 units (U) of Incobotulinumtoxin A (Xeomin, commercial lots 150070 and 154617, Merz GmbH) diluted with 0.9% saline to a concentration of 100 U/ml, or placebo (0.9% saline) divided between the muscles chosen for injection. Doses administered to each muscle were consistent with doses used in other upper limb spasticity studies [11], [14]–[16]. The placebo and active drug were identical in packaging and appearance. Injection sites within the muscle were determined according to surface landmarks for motor end points as specified in a standard textbook of neuroanatomy [17]. Electromyography was used to guide muscle localization.

#### Physical Therapy

The original design of the study included a physical therapy protocol. All study participants were intended to receive daily stretching and passive range of motion exercises in the injected limb starting the day following injections, occurring 5 days/week (20 minutes/session), and continuing throughout the study period (32 weeks). The exercise regimen administered would have consisted of a program designed to maintain muscle length, facilitating gentle movements of joints to prevent contractures and permanent shortening of muscles [18]. However, prior to enrollment of the first patient, new data became available (personal communication and subsequent study publication [19]), to suggest that passive range of motion exercise was not beneficial in the treatment of paratonia so this component of the intervention was not implemented.

### Outcome Measures

#### Assessments

At baseline and at 2, 6, 12, 16, 18, 22, 28, and 32 weeks, two blinded raters (a research assistant and a kinestheologist) at the bedside conducted assessments during morning care carried out by the professional caregiver. Each assessment was videotaped and reviewed by the two raters. Each assessment included all outcome measures. Assessments were performed two mornings per week/coordinated with the professional caregivers schedule and were at the same time on the two-assessment days/week but could vary week to week. Days of week were determined according to the availability of the caregiver. Scores were averaged over two-days/assessment week to account for the potential variability of the paratonia.

### Primary outcome

#### Carer Burden Scale

The Carer Burden Scale (CBS) is a function-related burden scale that addresses cleaning the palm, cutting the fingernails, dressing, and cleaning under the armpit in one limb. It uses a 5-point Likert scale from 0 (no difficulty) to 4 (cannot do the task) and then the score for each item is summed to provide a total CBS score (0–16) [14]. The original scale has a total score of 16. We removed fingernail cutting, as cutting fingernails was not consistently part of daily morning care in this population reducing the total score to 12. We also revised the scoring of armpit cleaning and palm cleaning into 2 questions each, 1 for each side, (individuals could receive injections in both limbs depending on impact of paratonia on care). This added two questions with a range of 0–4, adding up to 8 points, for a total score is 20 points. As such, we use the term “modified CBS” to clarify this is distinct from the original scale. The modified CBS (mCBS) was scored by the two raters who came to consensus on each sub-score based on observation of difficulty of caregiver to provide care.

### Secondary Outcome Measures

While our primary outcome of interest was change in mCBS, we also collected information on several secondary outcome measures which were subjected to exploratory analysis for hypothesis generation.

#### Joint angle measurement

Given the fluctuating nature of paratonia, using a static scale for increased tone due to spasticity, such as the Modified Ashworth Scale (MAS) [11], [20] is not suitable. As there is no validated, responsive scale to measure severity of paratonia, joint angle measurements reflecting range of motion, based on average measurements conducted at two separate time points in a week, were used as a surrogate measure for severity of paratonia. The two raters worked together to determine the joint angle with one rater maximally extending the limb and the other rater applying the goniometer. As patients varied in terms of pattern of clinically significant involuntary postures that interfered in care, only the limbs that met these criteria clinically had formal PROM measurements recorded of those specific postures (eg extension of elbow, flexion of wrist etc).

#### Global Assessment of Functional Status and Visual Analogue Scale of Caregiver Perception of Ease of Care

At each assessment, the overall response to treatment was evaluated by professional caregivers using the Global Assessment Scale (GAS) [21] of impression of change in difficulty of administering care. This scale estimates improvement or worsening compared to pre-treatment status and therefore, is not measured at baseline. It is anchored from −4 = very marked worsening (equating to increased stiffness interfering with the provision of care), to 0 = unchanged, to +4 = complete abolishment of difficulty (equating to absence of stiffness interfering in care). GAS was recorded twice (over two days) and responses were averaged for each assessment week. However, after study began, it became apparent that caregivers often changed from week to week and these impressions were provided by different caregivers who may not have had a baseline to compare to over time as a different caregiver responded in a previous assessment.

A Visual Analogue Scale (VAS) [22] of the caregiver’s perception of ease of care was also completed by the professional caregivers. The VAS consists of a 100 mm line with anchors of 0 “giving care to the residents is very difficult” and 100 “giving care to the residents is very easy”; scores represent the measured distance between 0 and the mark made by the caregiver on the line. VAS was recorded twice (over two days) and responses were averaged for each assessment week.

#### Pain Assessment

The Pain Assessment in Advanced Dementia (PAINAD) instrument [23] was used to assess whether treatments reduce pain in patients when having morning care performed. The PAINAD scale consists of 5 items (breathing, negative vocalization, facial expression, body language, and consolability) scored on a 0–2 point scale and then summed to arrive at a total score (range 0–10). This score was averaged over two days of assessments/week. Study personnel recorded the PAINAD score while observing morning care administered by the professional caregiver.

#### Time to Perform Care

Time to perform hygiene and dressing were determined with video recording analysis prior to and following injections. Specifically, time in minutes to perform individual goals (dressing (upper body), cleaning under arm, and cleaning palm; all tasks involved in the CBS) were timed by study personnel observing the professional caregiver. The videos were subsequently reviewed by the two raters. A stop watch was used to only include time specifically taken to perform specific task. The time determined following review of videos was averaged over 2 days of assessments.

### Safety

The study investigators assessed adverse events at each assessment including questions regarding swallowing impairments or breathing difficulty. A serious adverse event was defined as an event that was fatal, life-threatening, disabling or requiring hospitalization. An adverse event was any event that was reported by the study subject’s caregivers to have occurred during the study period.

### Statistical Analysis

As this was conceptualized as a pilot study, 10 subjects were chosen *a priori*, for provided statistical power of 70–80% to detect mean changes between baseline and 6 weeks of 0.5–1.5 points on the primary outcome of interest (i.e., the mCBS 5-point Likert scale). The range of estimates of statistical power reflects different assumptions regarding standard deviation (SD) of measurements (SD 0.5, 1, 1.5, or 2). The crossover design was anticipated to permit detection of clinically meaningful changes in measurement with treatment even with a relatively small sample size.

While analysis of variance (ANOVA) is often used for the analysis of crossover trial data, we utilized mixed modeling techniques [24] in order to utilize all available information collected on study subjects during intervention and control periods. Mixed modeling was also used to address between-study heterogeneity within and between patients, regarding paratonia severity, doses injected, what muscles were injected, and caregiver variability, through the introduction of patient-specific random effects (i.e., random intercepts). Treatment effect was assumed present by 6 weeks post-injection consistent with other studies [11], [12], [14], [15], [25]. The likelihood-ratio test was used to determine whether treating subjects as a random effect was necessary, and the Bayesian information criterion was calculated to help determine model fit. ANOVA was utilized to assess the efficacy of treatment with consideration of patient factors, sequence, sequence-by-treatment interaction, period, and period-by-treatment interaction effects. To account for the fluctuating nature of paratonia severity, measurements were performed twice (over a period of two days) with mean values from the two measurements used to assess outcomes. This provided an opportunity to evaluate reliability of measures, which was performed through calculation of intra-class correlation coefficients. For purposes of hypothesis generation, secondary outcome measures were subjected to exploratory statistical analysis.

All analyses were performed in Stata version 12.0, (Stata Corp., College Station, TX).

## Results

### Subjects

The study began in January 2011 and ended in March 2013. Ten subjects were enrolled in the study. Subject baseline demographic information is included in **Table 1** **.** Subjects were randomly allocated to each treatment sequence. There were no significant differences in the characteristics of the subjects in the two treatment sequence allocations (**Table 2**). One subject in the Incobotulinumtoxin A – placebo sequence (Subject 4) died during week 10 of the study. Data on this subject was only collected and included up to Week 6.

**Table 1:** Subject demographics.

Subject	Age (yrs)	Gender(M/F)	Diagnosis	DiseaseDuration (yr)	PAI	Total DoseIncobotulinumtoxin A (U/ml)
1	84	F	AD*	9	3	200
2	96	F	AD	11	4	300
3	86	F	FTD§	5	3	300
4¶	88	F	AD	11	3	300
5	75	F	AD	8	3	240
6	93	M	AD	8	3	300
7	88	F	AD	8	3	175
8	69	F	AD	6	3	300
9	89	F	AD	12	3	175
10	85	F	AD	10	3	300
**Mean** ± **SD**	85.30±8.00			8.80±2.25	3±0.47	259±55.82

*Alzheimer’s disease.

§ Fronto-temporal dementia.

¶ Died week 10.

PAI: Paratonia Assessment Instrument.

**Table 2:** Subject characteristics.

Characteristic	Sequence 1 (Placebo-Incobutulinumtoxin A) [N = 5]	Sequence 2 (IncobotulinumtoxinA-Placebo) [N = 5]	P value
Mean age, *yr* (range)	84.6 (69–96)	86.0 (75–93)	0.800
Female, *n* (%)	5 (100)	4 (80)	0.347
Diagnosed AD, *n* (%)	5 (100)	4 (80)	0.347
Disease duration, mean±SD	9.60±2.30	8.00±2.12	0.286
PAI, mean±SD	3.20±0.45	3.00±0.45	0.195
Baseline mCBS-Total, mean±SD	5.40±3.44	7.30±4.66	0.209
Total Incobotulinumtoxin A dose, mean U/ml±SD	255.00±62.25	263.00±55.63	0.836

PAI: Paratonia Assessment Instrument.

mCBS: modified Carer Burden Scale.

### Doses and Injections

The distribution and doses of Incobotulinumtoxin A injected are presented in **Table 3** **.** **S1** **Table** provides the specific muscles injected in each subject.

**Table 3:** Muscles and doses injected.

Muscle	Incobotulinumtoxin A units (U),Median (min, max)	Patients injected n (%)
BB	50 (25,100)	10 (100%)
BR	45 (25,100)	5 (50%)
PM	90 (50,125)	8 (80%)
FDS	75 (50,90)	7 (70%)
FDP	35 (20,50)	2 (20%)
FPL	15 (10,25)	3 (30%)
Tricep	50 (25,50)	6 (60%)
Lumbricals	35 (30,40)	2 (20%)
FCU	25 (25,25)	1 (10%)
OP	17.5 (15,20)	2 (20%)

BB = Biceps Brachii, BR = Brachioradialis, PM = Pectoralis Major, FDS = Flexor Digitorum Superficialis, OP = Opponens Pollicis, FDP = Flexor Digitorum Profundus, FPL = Flexor Pollicis Longus, FCU = Flexor Carpi Ulnaris.

### Primary outcome

#### Carer Burden Scale

**Figure 2:**
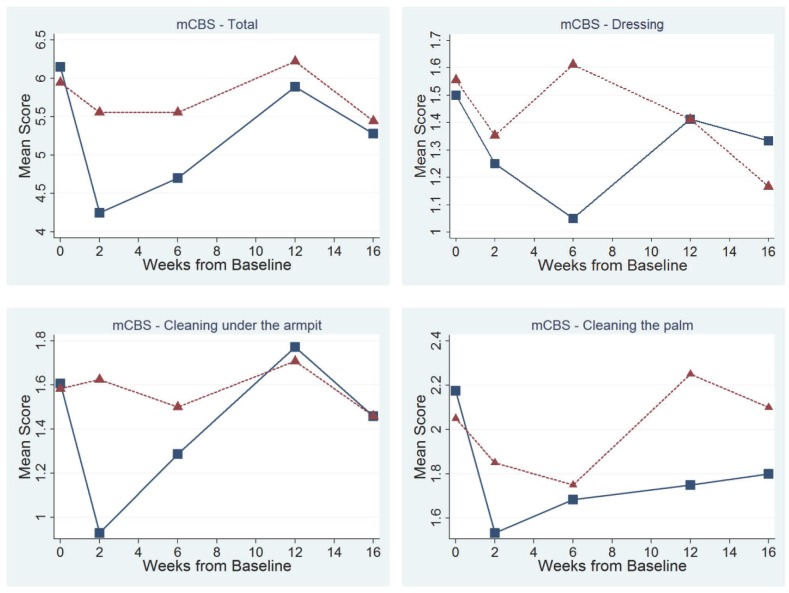
mCarer Burden Scale (mCBS) in each subject treated with Incobotulinumtoxin A (□) and Placebo (Δ).

Graphical evaluation of mCBS scores identified reduced (improved) mean total scores and sub-scores during the interval 2 to 6 weeks (peak effect) following injection with toxin (**Fig. 2**) with gradual return to baseline by 12 weeks. Mixed effects regression models evaluating treatment effects from 2 to 6 weeks post-injection identified a significant Incobotulinumtoxin A treatment effect for the mCBS total score and for the dressing and cleaning under the arm subscales (**Table 4**). Incobotulinumtoxin A reduced overall mCBS score by 1.11 (p = 0.02); dressing sub-scores were reduced by 0.36 (p = 0.004); and scores for cleaning under the left and right armpits were reduced by 0.50 (p = 0.03) and 0.41 (p = 0.03) respectively. There was reduction in cleaning of the left and right palms of the hand 0.25 (p = 0.4), and 0.05 (p = 0.8) respectively, but this did not reach statistical significance. By contrast, ANOVA comparing baseline to 6-week effects found no significant differences between treatment and placebo in mCBS total score (p = 0.2), though significant improvement was present in the dressing component of the mCBS (p = 0.01). No period or sequence effects were detected. Models’ fits were improved without substantial loss of parsimony with the incorporation of subject random effects, as demonstrated by reductions in the Bayesian information criterion, suggesting that between-individual heterogeneity in disease and response was an important consideration in explaining Incobotulinumtoxin A treatment effects (**S2 Table**).

**Table 4:** Mixed model outcomes of mCBS, PROM, PAINAD, GAS, VAS and time to perform care at 2 and 6 weeks after treatment with Incobotulinumtoxin A.

OUTCOME MEASURE	N	TREATMENT EFFECT (95% CI)	P-VALUE
**mCARER BURDEN SCALE**			
Total score	10	–1.11 (–2.04 to −0.18)	**0.02**
Dressing	10	–0.36 (–0.59 to −0.12)	**0.004**
Cleaning under arm (left)	7	–0.50 (–0.96 to −0.04)	**0.034**
Cleaning under arm (right)	7	–0.41 (–0.79 to −0.04)	**0.030**
Cleaning palm (left)	5	–0.25 (–0.78 to 0.28)	0.4
Cleaning palm (right)	6	–0.05 (–0.47 to 0.38)	0.8
**RANGE OF MOTION**			
Elbow extension (left)	6	27.67 (13.32 to 42.02)	**<0.001**
Elbow extension (right)	8	22.07 (9.76 to 34.39)	**<0.001**
Elbow flexion (left)	4	9.94 (–0.32 to 20.19)	0.06
Elbow flexion (right)	2	–5.81 (–16.23 to 4.61)	0.3
Finger extension (left)	5	19.85 (3.25 to 36.45)	**0.02**
Finger extension (right)	6	23.56 (3.46 to 43.67)	**0.02**
Shoulder abduction (left)	7	11.92 (5.46 to 18.38)	**<0.001**
Shoulder abduction (right)	7	8.58 (3.73 to 13.43)	**0.001**
Thumb abduction/extension (left)	3	17.75 (–0.79 to 36.29)	0.06
Thumb abduction/extension (right)	2	–0.50 (–9.68 to 8.68)	0.9
**OTHER SECONDARY** **MEASURES**			
Pain Assessment in Advanced	10	–0.09 (–0.70 to 0.51)	0.8
Dementia Scale (PAINAD)			
Global Assessment Scale (GAS)	10	0.53 (–0.07 to 1.13)	0.08
Visual Analogue Scale (VAS)	10	5.30 (–4.64 to 15.23)	0.3
Cleaning time	10	–4.50 (–25.85 to 16.84)	0.68
Dressing time	10	–3.99 (–13.03 to 5.06)	0.29

### Secondary outcomes

#### Passive Range of Motion

**Figure 3:**
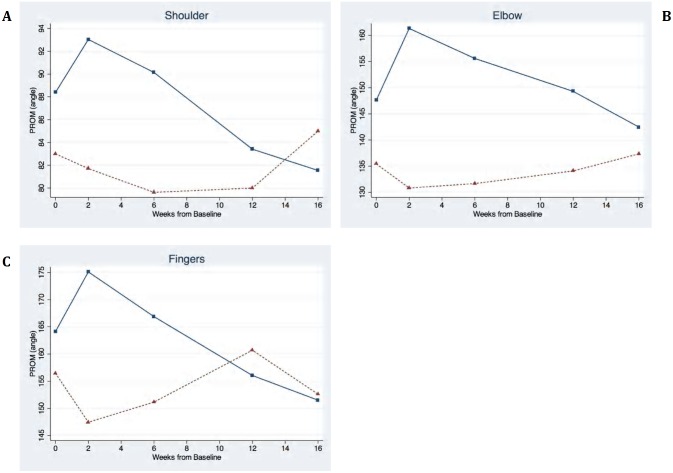
Passive range of motion (PROM) following Incobotulinumtoxin A injections (□) and Placebo (Δ) treatments for: A) Shoulder B) Elbow C) Fingers.

Significant improvement in passive range of motion (PROM) as represented by increased joint angles was detected for most joints with mixed effects models (**Fig. 3**): Left and right elbow extension increased by 27.67 degrees (p<0.001) and 22.07 degrees (p<0.001), while left and right finger extension increased by 19.85 degrees (p = 0.02) and 23.56 degrees (p = 0.02). Improvements were also seen in the left and right shoulder abduction by 11.92 degrees (p<0.001) and 8.58 degrees (p = 0.001).

#### GAS and VAS of Burden of Care, PAINAD

No significant treatment effects were found for the GAS, the VAS of caregiver perceived burden of care, or the PAINAD.

#### Time to perform care

Though time taken to perform care was recorded at each assessment, the protocol for provision of morning care was not standardized and there was significant variability in technique and efficiency of administration of morning care. Some caregivers worked more slowly than others reflecting their abilities and energy levels rather than patient related factors. Time to perform care is reported in **Table 4**.

### Reliability

All measures used in this study appeared reliable based on estimation of intra-class correlation coefficients (ICC). ICC were high for the CBS, with near-perfect reproducibility for both the total score, and cleaning and dressing sub-score, and joint angle measurements between day 1 and day 2 of assessments reflecting high reliability for ratings performed by study personnel. GAS, VAS for caregiver perceived burden of caregiving, and PAINAD also demonstrated good reliability within each week (**S3 Table**).

### Safety

There were no adverse drug reactions reported by the caregivers of the study participants. One subject (Subject 4) died 10 weeks into the study (Incobotulinumtoxin A-placebo sequence). At the time of the patient’s death, treatment was unblinded to determine exposure to Incobotulinumtoxin A or placebo. The subject died of heart failure. This was deemed unrelated to Incobotulinumtoxin A injections after a review by the treating physicians, study investigators and by the pharmacovigilance drug safety group at Merz GmbH.

## Discussion

To our knowledge, this study represents the first clinical randomized, double blind, placebo-controlled trial of Botulinum toxin administration for paratonic rigidity in persons with dementia; indeed, we believe this is the first clinical trial of any type for this indication. Though this is a small pilot study, the results suggest that in elderly, dependent, cognitively impaired individuals with increased muscle tone due to paratonia, Incobotulinumtoxin A is an intervention that provides meaningful reduction in caregiver burden. In exploratory analyses related to secondary outcomes, we also identified significant benefits to patients related to increased range of motion of affected limbs, an effect that could have the downstream benefit of preventing contractures. This is a pilot study, and these findings await confirmation in larger studies, but statistical significance in the context of modest sample size does underline the large effects observed in this group of patients.

We found Incobotulinumtoxin A injections to be safe in this population of elderly individuals with advanced dementia; most other studies of Botulinum toxin have included younger individuals [11], [14], [15], [25]. There has been concern that known risks such as swallowing impairment or breathing difficulties would be enhanced in this population, but in this (small) study we did not identify adverse drug reactions. The single fatality during the trial occurred in an 88 year-old individual and careful investigation by treating physicians (independent of study) revealed no relationship between his death and Incobotulinumtoxin A therapy.

The potential to reduce caregiver burden in this population is an important health and health-economic objective. In people with advanced cognitive impairment and total dependence on caregivers for all aspects of care, caregiver burden has been demonstrated to be a strong predictor of health-related quality of life [26]. It is commonly accepted that Botulinum toxin injections reduce spasticity and increase range of motion in the upper-limb of post-stroke ambulatory patients, but a recent randomized trial identified similar results in long-term care residents with post-stroke upper limb spasticity [25]. In that population, there was a significant reduction in both muscle tone and (consequently) caregiver burden as measured by the CBS as the primary outcome measure [25]. While our study subjects experienced increased tone due to paratonia, not spasticity, long-term immobility resulted in similar functional consequences for caregivers. Though the underlying pathophysiology of increased tone was different (spasticity vs. paratonia), the impact on caregiver burden as a result of treatment was similar.

We identified a non-significant trend towards reduced pain in some subjects using the PAINAD scale. There was a similar absence of a significant effect in the study by Lam et al [25] leading those investigators to question the validity of the PAINAD scale in this type of population. However, insufficient power or sensitivity of the scale to capture change in this type of population may be more likely explanations for the lack of significant change demonstrated in our study.

In other secondary outcomes in this trial, we did not identify significant changes in the GAS and VAS of caregiver assessment of difficulty of provision of care. The lack of significant improvements in these measures compared with improvements in mCBS may reflect the fact that professional caregivers completed these measures, which changed in some cases from week to week, in contrast to the mCBS, which was rated consistently by the same trained study staff. As such, though there was good intra-rater reliability between assessments on day 1 and 2 as staffing was consistent for one-week intervals, professional caregivers changed weekly. The GAS is based on comparison of the previous weeks assessment (as opposed to independent observations like the other outcome measures), which made the results difficult to interpret and put into question the validity of the responses to these items. Likewise the VAS rates current ease of care but to assess its significance, its needs to be compared to a previous assessment. If different caregivers are providing this impression, it is impossible to determine magnitude of change given subjective nature of this outcome measure and the variability of respondents.

This pilot study was conceived in part, to determine feasibility of the protocol and of the battery of assessments that could eventually be used in a phase III trial if further study was warranted. For example, experience gathered during the trial demonstrated that measurement of time to provide care, while an important and valuable piece of information that could be linked to health economics, was not reliably captured with this protocol. As for each patient several caregivers provided care, “noise” was introduced, as inter-individual differences in how they went about providing care may have washed out the patient factors that contribute to the time to provide care. For this variable to be accurate again, the same caregiver needs to provide care at all time points, and the way care is provide needs to be standardized, such that changes in the patient impacting the time to provide care are captured.

In addition to the already described limitations, other limitations to consider include the small sample size of the study, which was defined a priori as appropriate for a pilot study. While the use of mixed effects models allowed us to utilize all available measurements, and to adjust for between-subject heterogeneity, it is nonetheless likely that we lacked power for stable estimation of treatment effects, and some of the negative results described above may be due to type II error. Generalizability of the study may also be limited as a result of the fact that 9/10 subjects carried the diagnosis of Alzheimer’s disease despite inclusion criteria including other forms of cognitive impairment and 9/10 subjects were female. Further limitation was the fact that the Caregiver Burden Scale (CBS), while used as a primary outcome measure in numerous published trials of Botulinum toxin efficacy in a stroke population, has not been formally validated for patients with paratonia. We modified this scale to reflect the bilateral nature of paratonia as the scale was originally envisioned only for those with stroke which typically is limited to unilateral disability and as such the modifications we made to the scale have not been validated. Currently, there are no valid outcome measures specifically examining caregiver burden in this population. Future study would need to consider modifying and validating the CBS for patients with paratonia.

We did not collect cost-related data during this trial, and this is an important aim for future work. While this treatment is expensive, it is possible that this cost is justified by reduction in caregiver burden.

In conclusion, consistent with the growing consensus on the use of Botulinum toxin use to prevent spasticity post-stroke, we found that in this small number of patients with advanced dementia who experienced paratonic rigidity, administration of Botulinum toxin, facilitated provision of care by preserving range of motion of affected limbs. If these results are replicated on a larger scale, the use of Botulinum toxin has potential to improve health-related quality of life in dependent, cognitively impaired individuals. Given the rising economic and social burden of dementia care [27] these promising results should be followed up with a large, well-designed clinical trial.

## Supporting Information

S1 Table **Muscles injected in individual patients.**
(DOCX)

S2 Table **Assessing the necessity of subjects random effect.**
(DOCX)

S3 Table **Reliability in scoring between day 1 and 2.**
(DOCX)

S1 Protocol **Trial protocol.**
(DOC)

S1 CONSORT Checklist **CONSORT Checklist.**
(PDF)
