# Supplementary material for: A Randomized, Placebo Controlled Pilot Trial of Botulinum Toxin for Paratonic Rigidity in People with Advanced Cognitive Impairment
Source: PLoS One. 2014 Dec 23;9(12):e114733. doi: 10.1371/journal.pone.0114733 (PMC4275182; doi:10.1371/journal.pone.0114733)
Supplement: S1 Table — Muscles injected in individual patients. (DOCX) [file pone.0114733.s001.docx]

| Subject | Muscle Injected | Right  (U) | Left  (U) | Total  (U) |
| --- | --- | --- | --- | --- |
| 1 | BB  BR | 50  50 | 50  50 | 200 |
| 2 | PM  Tricep  FDS  OP  Lumbricals (3,4,5)  BB  BR | 40  50  50  15  30  -  - | 40  -  -  -  -  50  25 | 300 |
| 3 | Tricep  FDS  Lumbricals (3,4,5)  BB  BR  OP  FPL | -  45  20  45  45  10  - | 50  45  20  -  -  10  10 | 300 |
| 4 | PM  Tricep  BB  FDS  FDP | 75  .  25  50  50 | 50  50  -  - | 300 |
| 5 | PM  FDS  Bicep  Tricep | 50  25  -  25 | 50  40  50  . | 240 |
| 6 | BR  BB  FDS  PM  FPL | -  25  25  -  - | 50  75  50  50  25 | 300 |
| 7 | PM  BB  BR  Tricep | 50  50  25  - | -  -  -  50 | 175 |
| 8 | FDS  PM  BB  FCU | 25  50  50  - | 50  50  50  25 | 300 |
| 9 | PM  BB | 50  50 | 25  50 | 175 |
| 10 | PM  BB  FDS  FPL FDP Tricep | 50  50  -  -  -  - | 50  -  75  15  20  40 | 300 |

**Muscles injected in individual patients: Supporting information Table S1**

BB=Biceps Brachii, BR=Brachioradialis, PM=Pectoralis Major, FDS=Flexor Digitorum Superficialis, OP=Opponens Pollicis, FDP=Flexor Digitorum Profundus, FPL=Flexor Pollicis Longus, FCU=Flexor Carpi Ulnaris
