# Supplementary material for: A Randomized, Placebo Controlled Pilot Trial of Botulinum Toxin for Paratonic Rigidity in People with Advanced Cognitive Impairment
Source: PLoS One. 2014 Dec 23;9(12):e114733. doi: 10.1371/journal.pone.0114733 (PMC4275182; doi:10.1371/journal.pone.0114733)
Supplement: S2 Table — Assessing the necessity of subjects random effect. (DOCX) [file pone.0114733.s002.docx]

**Assessing the necessity of subjects random** **effect: Supplementary Information Table S2**

| OUTCOME MEASURE | LIKELIHOOD-RATIO TEST P-VALUE | BIC (SUBJECTS RANDOM EFFECT) | BIC (NO RANDOM EFFECT) |
| --- | --- | --- | --- |
| CARER BURDEN SCALE |  |  |  |
| Total Score | <0.001 | 67.4 | 85.1 |
| Dressing | <0.001 | 70.1 | 93.0 |
| Cleaning under arm (left) | 0.03 | 67.3 | 68.6 |
| Cleaning under arm (right) | 0.02 | 57.2 | 59.5 |
| Cleaning palm (left) | 1 | 48.7 | 45.7 |
| Cleaning palm (right) | 0.02 | 52.6 | 54.9 |
| RANGE OF MOTION |  |  |  |
| Elbow extension (left) | 0.002 | 230.7 | 237.2 |
| Elbow extension (right) | 0.05 | 276.9 | 277.4 |
| Elbow flexion (left) | 0.03 | 138.1 | 140.1 |
| Elbow flexion (right) | 0.017 | 51.2 | 55.1 |
| Finger extension (left) | 1 | 186.4 | 183.4 |
| Finger extension (right) | 0.91 | 214.9 | 211.8 |
| Shoulder abduction (left) | 0.1 | 204.3 | 203.7 |
| Shoulder abduction (right) | <0.001 | 194.5 | 203.6 |
| Thumb abduction/extension (left) | 0.2 | 114.7 | 114.1 |
| Thumb abduction/extension (right) | 0.5 | 62.7 | 61.1 |
| OTHER SECONDARY MEASURES |  |  |  |
| Pain Assessment in Advanced  Dementia Scale (PAIND) | <0.001 | 137.6 | 152.8 |
| Global Assessment Scale | 0.3 | 122.9 | 120.4 |
| Visual Analogue Scale | 0.006 | 343.0 | 347.0 |
