# Supplementary material for: A Randomized, Placebo Controlled Pilot Trial of Botulinum Toxin for Paratonic Rigidity in People with Advanced Cognitive Impairment
Source: PLoS One. 2014 Dec 23;9(12):e114733. doi: 10.1371/journal.pone.0114733 (PMC4275182; doi:10.1371/journal.pone.0114733)
Supplement: S3 Table — Reliability in scoring between day 1 and 2. (DOCX) [file pone.0114733.s003.docx]

**Reliability in scoring between day 1 and 2 Supporting Information Table S3**

| OUTCOME MEASURE | N | ICC* |
| --- | --- | --- |
| CARER BURDEN SCALE (CBS) |  |  |
| Total Score | 10 | 0.935 |
| Dressing | 10 | 0.918 |
| Cleaning under arm (left) | 7 | 0.859 |
| Cleaning under arm (right) | 7 | 0.814 |
| Cleaning palm (left) | 5 | 0.819 |
| Cleaning palm (right) | 6 | 0.893 |
| RANGE OF MOTION |  |  |
| Elbow extension (left) | 6 | 0.955 |
| Elbow extension (right) | 8 | 0.829 |
| Elbow flexion (left) | 4 | 0.925 |
| Elbow flexion (right) | 2 | 0.820 |
| Finger extension (left) | 5 | 0.803 |
| Finger extension (right) | 6 | 0.691 |
| Shoulder abduction (left) | 7 | 0.900 |
| Shoulder abduction (right) | 7 | 0.920 |
| Thumb abduction/extension (left) | 3 | 0.937 |
| Thumb abduction/extension (right) | 2 | 0.716 |
| OTHER SECONDARY MEASURES |  |  |
| Pain Assessment in Advanced  Dementia Scale (PAINAD) | 10 | 0.927 |
| Global Assessment Scale (GAS) | 10 | 0.826 |
| Visual Analogue Scale (VAS) | 10 | 0.845 |

*ICC: intraclass correlation co-efficient
