# Supplementary material for: A Randomized, Placebo Controlled Pilot Trial of Botulinum Toxin for Paratonic Rigidity in People with Advanced Cognitive Impairment
Source: PLoS One. 2014 Dec 23;9(12):e114733. doi: 10.1371/journal.pone.0114733 (PMC4275182; doi:10.1371/journal.pone.0114733)
Supplement: S1 Protocol — Trial protocol. (DOC) [file pone.0114733.s004.doc]

**Botulinum toxin for patients with cognitive impairment and paratonia**

**GSK-01**

**Project Summary**

**Background:** Dementia prevalence in Canada is high and projected to increase in coming decades. The economic consequences of dementia are profound with current estimates of $15 billion/yr in Canada. In addition to reduced mental capacity, these cognitively impaired, dependent individuals develop motor impairments and become increasingly immobile. The combination of increased muscle tone due to the degenerative process along with prolonged immobility, may lead to fixed contractures that cause the individual pain and discomfort, put them at risk for skin breakdown, and interfere with the ability to receive care. Preliminary work has suggested that in those with increased tone that interferes with the provision of care, administration of Botulinum toxin to select muscle groups, reduces time taken to administer hygiene, decreases effort and exertion by staff, and reduces the discomfort of the patient.

**Objective:** Perform a pilot study to assess the impact of Botulinum toxin type A on muscle tone, time to provide care, and carer burden in individuals who are cognitively impaired and fully dependent.

**Methods:** *Trial design:* The study will be a blinded, placebo-controlled, randomized trial with a crossover design with two treatment cycles of 16 weeks each. Following baseline assessment and injections, repeated assessments will take place at 2, 6, 12 and 16 weeks post injections.

*Intervention*: Subjects will be randomly allocated to receive either active Botulinum toxin injections at baseline or 16 weeks or control sterile saline injections at the alternate injection period. The patient, professional caregiver and study investigators involved in assessments will be blinded to treatment allocation.

*Setting*: The trial will take place at a spasticity management clinic with infrastructure (personnel, equipment, and space) and experience for safe and efficacious administration of Botulinum toxin injections.

*Subjects:* 10 fully dependent patients with cognitive impairment that have increased upper extremity tone interfering with the provision of care.

*Outcome measures*: The primary outcome measure will be the Carer Burden Scale assessing ease of administering care that includes aspects of providing hygiene and dressing. Secondary outcome measures will include the Modified Ashworth Scale (MAS) objectively measuring increased tone, time taken to perform tasks (morning care routine) before and after treatment, and the caregiver Visual Analogue Scale (VAS) assessing perception of ease of care-giving, nurse-investigator and professional and caregiver Global Assessment Scale (GAS) assessing overall response to treatment. The Pain Assessment in Advanced Dementia (PAINAD) instrument will be used to assess whether treatments reduce pain in patients when having morning care performed. Video-recordings of morning care routines prior and following treatments will be reviewed and compared to ensure reliability. Additionally, the caregiver will be questioned in an open-ended format to determine what additional factors contributed to relevant changes in the care of patients as a result of the injections that may not have been captured with quantitative measures.

**Statistical Analysis:** The proposed crossover design warrants the use of mixed regression modeling techniques in order to isolate the effect of Botulinum toxin, relative to placebo on the described outcomes. Treatment effects will be defined as the change in relevant scores from baseline to 6 weeks, and 16 to 22 weeks (i.e., change from baseline until 6 weeks post-treatment or placebo).

**Conclusions:** In these times of fiscal constraint, difficult decisions regarding resource allocation to optimize patient care need to be made. An intervention with the capacity to reduce care burden, improve standard of care, and potentially increase patient and caregiver quality of life, would be an attractive strategy. Preliminary experience suggests that Botulinum toxin injections may result in this effect. This pilot study will assess the feasibility of conducting a larger study that will evaluate the impact and cost-effectiveness of reduction of tone in cognitively impaired, fully dependent individuals.

.

**Proposal**

**Objectives:**

Perform a pilot study to assess the impact of Botulinum toxin type A on muscle tone, time to provide care, and carer burden in individuals who are cognitively impaired and fully dependent.

**Background:**

Dementia is highly prevalent in Canada: an estimated 1.5% of all Canadians live with dementia (nearly 500,000 people). A new case is diagnosed approximately every 5 minutes. It is projected that by 2038, due to population ageing, the prevalence of dementia will be 2.8% with a new case being diagnosed every 2 minutes (1). A 1994 Canadian study of health and aging estimated that the prevalence of dementia in those over the age of 65 was ~ 8% with approximately 50% of these individuals lived in long-term care settings (2). Further, 62% of long-term care residents met criteria for dementia (3).

The economic consequences of this disease are profound both in terms of financial and caregiver burden. The current costs of dementia to the Canadian economy are estimated at $15 billion per year, and by 2038 that cost is projected to be $153 billion (1). A study assessing the economic burden of Alzheimer’s disease calculated the costs of caring for an institutionalized, severely cognitively impaired person to be approximately $2.5 billion in the US in the year 2000 with a projected tripling of these costs by 2040 (4). In 2000, Canada spent a remarkable 1.06% of its GDP to finance costs of long-term institutionalized care (5).

In addition to cognitive deterioration, dementia is often accompanied by motor disability that amplifies caregiver burden. Immobility in demented individuals is due in part to paratonia, a form of increased tone first observed by Dupre, in 1910, that he characterized as “an inability to relax muscles in the setting of cognitive impairment” (6). Postulated to originate in the central nervous system, paratonia exerts its effects by increasing muscle resistance. In advanced stages of dementia, it is associated with reduced mobility and the development of fixed postures (contractures). Paratonia has been estimated to be present in 5% of those with mild cognitive changes and 100% in those with advanced dementia (7-10).

It is commonly accepted that once contractures have developed, medical treatments are futile and for these unfortunate individuals, families and care-givers, management strategies must be found that take into account these limitations. The consequences of fixed postures include difficulty accessing body regions to provide hygiene which increases caregiver burden, and reduces quality of provision of care resulting in consequences such as infection or skin breakdown. Additionally, the individual receiving care may experience discomfort and pain as a result of efforts to manipulate fixed limbs.

**Rationale for Study:**

Botulinum toxin type A (Botox™, Xeomin™) is an exotoxin produced by the bacterium *Clostridium botulinum*. It can be used as a therapeutic intervention to selectively weaken skeletal muscle in a dose-related manner by impairing the release of acetylcholine, a neurotransmitter, at the neuromuscular junction (11). While commonly used for cosmetic purposes to reduce wrinkles, it has found therapeutic indications in people with abnormal muscle tone. Currently, it is the approved treatment of choice in Canada for conditions including blepharospasm, strabismus, hemifacial spasm, cervical dystonia, spasmodic dysphonia, and foot deformity in cerebral palsy patients. Botulinum toxin has also been proven safe and effective for the treatment of post-stroke spasticity. In stroke patients with spasticity, it reduces tone and improves the capacity to independently perform activities of daily living (ADLs)(12); in those requiring full care due to disability following stroke, administration of Botulinum toxin has been shown to reduce caregiver burden (13). Injections are well tolerated and are effective for approximately 3 months. Side-effects are minimal due to the fact that Botulinum has local and not systemic effects, with none of the systemic consequences that limit the use of orally administered agents for tone reduction. For patients with spasticity due to multiple sclerosis, the addition of a physical therapy regimen has been shown to further augment the effects of Botulinum toxin on tone reduction with improved function (14).

There is a growing consensus that this intervention should be applied early post-stroke, as a first-line treatment to reduce or prevent disability that may occur as a result of prolonged immobility (15). By reducing increased tone due spasticity following stroke, rehabilitation is facilitated, preventing permanent contractures and related sequelae. Similarly, it could be extrapolated that patients with paratonic rigidity may experience benefit from early administration of Botulinum toxin, which should facilitate provision of care by preserving range of motion, thereby delay onset of contractures.

To date, studies have not been performed to evaluate the efficacy of Botulinum toxin injections for paratonia. However, the profoundly detrimental impact on patient and caregiver quality-of-life as a result of immobile limbs in individuals with dementia has prompted us to offer Botulinum toxin injections to a small cohort of patients evaluated for this indication at the Elkie Adler Spasticity Management Clinic. We have found that common postures that occurred in the arm and were amenable to improvement through Botulinum toxin injections included: adducted shoulder, flexed elbow, wrist, and fingers, and a thumb-in-palm deformity, similar to postures following stroke. Such postures interfere in the provision of care and/or result in pain or skin breakdown, and diminish quality of life for patients and caregivers. Administration of Botulinum toxin in these few patients resulted in increased ease of care, decreased time to perform care, improved skin integrity, and reports of reduction in caregiver burden and patient discomfort when hygiene and morning care (defined as the period of time between 7:00 am and 12:00 pm when caregivers are engaged in activities related to bathing, grooming, dressing and toileting(16)) were administered. As such, we hypothesize that dependent individuals with dementia and increased tone resulting in involuntary postures may benefit from treatment with Botulinum toxin injections.  We propose to conduct a pilot study to test the above hypothesis, and to refine the approach to Botulinum toxin use in this context, in preparation for subsequent prospective studies.  The ultimate objective of a phase III trial would be to prove that Botulinum toxin injections are efficacious, safe and cost-effective in reducing burden of care and improving standard of care for fully dependent, cognitively impaired individuals with increased tone interfering in care.

**Pilot study objectives**

*Primary Objective*:

1. Confirm proof of principle that paratonic rigidity and its consequences can be reduced with Botulinum toxin injections resulting in reduced caregiver burden.

*Secondary Objectives:*

1. Determine optimal time points for evaluation of efficacy in this patient
   population and whether this time period is sufficient for washout of Botulinum toxin effect
2. Determine most salient and sensitive outcome measures
3. Identify obstacles in data gathering
4. Determine feasibility of battery of assessments in this pilot study that will eventually be used in a future phase III study

**Methods**

*Trial design:*

We propose a randomized, double-blind, placebo-controlled, crossover trial design. All subjects will thus act as their own controls. There will be 2 cycles of injection: at baseline and at week 16. All subjects will be randomized to receive active Botulinum toxin injection or sterile saline injections and will receive the alternate substance at the subsequent injection. The investigator assessing outcome, the study subject and the professional caregiver (primary nurse) will be blinded to treatment allocation.

*Setting:*

The Elkie Adler Spasticity Management Clinic is composed of a multidisciplinary team of physiatrists, neurologists, physical and occupational therapists, clinical nurse specialists, a social worker and a kinesiologist. There is a seating Clinic and an assistive communication device program on-site. It is located at Baycrest, in Toronto, Ontario, a geriatric-focused institution with an affiliated retirement home, nursing home and chronic-care hospital, as well as outpatient specialty Clinics. The mandate of the Clinic is to provide a multi-disciplinary team approach to patients with disabilities in order to address every aspect of their activities of daily living and to enable them to optimize their full potential to live independently and in comfort.

*Participants:*

Ten individuals with severe cognitive impairment, requiring total care for basic activities of daily living (ADLs), and having increased tone in an arm, that is interfering with the provision of care will be included in the study. If bilateral limbs are affected, only one (the more severely affected) limb will be injected.

Subjects will be eligible for the study if they have a score ≥3 (considerable resistance to passive movement) on the Paratonia Assessment Instrument (PAI) in an affected limb, and which results in one or more of: (i) difficulty in provision of morning care; (ii) postures resulting in pain when flexing, extending or abducting the limb; or (iii) postures resulting in skin breakdown or resulting in pressure ulcers. Exclusion criteria will include the presence of an alternate etiology for increased tone (ie., parkinsonian syndromes manifesting rigidity, known strokes or other focal neurological deficits), fixed contractures of the affected limb (assessed clinically as no mobility on passive range of motion), or injection with Botulinum toxin in the preceding 6 months.

*Intervention:*

Muscles to be injected and exact doses injected will be determined based on the characteristics of the posture causing disability (amount of increased tone, size of muscle) using EMG-guided muscle exploration at the time of injection with an upper dose limit of 300U based on doses used in other upper-limb spasticity studies (12, 15, 17). Injection sites within the muscle will be determined according to surface landmarks for motor end-points as specified in a standard text of neuromuscular anatomy (18).

*Study procedure:*

**Randomization**

The choice of the first injection will be made by consulting a table of random numbers that will be determined by the pharmacy representative at Baycrest Hospital that will be part of the study team. The pharmacist will be responsible for the all allocation codes and preparation of the Botulinum toxin and placebo syringes, and distribute these accordingly to the treating physician.

**Blinding**

Botulinum toxin for injection will be prepared at the pharmacy by diluting Xeomin (Merz GmBH) with 0.9% saline to a concentration of 100 mouse units (MU)/mL. As the solution is colorless and odorless, it is indistinguishable from an equivalent volume of isotonic saline which will be used for the placebo injections. The syringes with solution, either placebo or Botulinum toxin A, will be sent to the Elkie Adler Spasticity Management Clinic labeled with the subject’s code. All the investigators at the Clinic, the subject and the professional caregiver will be blinded to whether a subject received drug or placebo during the entire study. A procedure to break code will be in place at the pharmacy in case of medical emergency.

**Physical Therapy**

All study participants will receive daily stretching and passive range of motion exercises in the injected limb starting the day following injections, occurring 5 days/week (20 minutes/session), and continuing throughout the study period (32 weeks). The exercise regimen administered will consist of a program designed to maintain muscle length, facilitating gentle movements of joints to prevent contractures and permanent shortening of muscles (14). The physical therapy regimen will be taught to the professional caregiver by a physical therapist at the Clinic. At every evaluation session, the physical therapy protocol will be reinforced by the study nurse.

**DEVIATION OF FINAL PROTOCOL FROM PROTOCOL SUBMITTED TO REB**

***Based on subsequent data that was published where it was clear that physical therapy in paratonia did NOT improve outcomes, this component of the protocol was not included (***[***Int Psychogeriatr.***](http://www.ncbi.nlm.nih.gov/pubmed/22185768) ***2012 May;24(5):834-44. doi: 10.1017/S1041610211002468. Epub 2011 Dec 20.Passive movement therapy in severe paratonia: a multicenter randomized clinical trial.*** [***Hobbelen JH***](http://www.ncbi.nlm.nih.gov/pubmed?term=Hobbelen JH%5BAuthor%5D&cauthor=true&cauthor_uid=22185768)***^1^,*** [***Tan FE***](http://www.ncbi.nlm.nih.gov/pubmed?term=Tan FE%5BAuthor%5D&cauthor=true&cauthor_uid=22185768)***,*** [***Verhey FR***](http://www.ncbi.nlm.nih.gov/pubmed?term=Verhey FR%5BAuthor%5D&cauthor=true&cauthor_uid=22185768)***,*** [***Koopmans RT***](http://www.ncbi.nlm.nih.gov/pubmed?term=Koopmans RT%5BAuthor%5D&cauthor=true&cauthor_uid=22185768)***,*** [***de Bie RA***](http://www.ncbi.nlm.nih.gov/pubmed?term=de Bie RA%5BAuthor%5D&cauthor=true&cauthor_uid=22185768)***.)***

**Outcomes**

Assessment of outcomes will occur at baseline/screening, 2, 6, 12 and 16 weeks following injections at baseline and 16 weeks. Week 6 and 22 have been chosen as the primary endpoints, as reduction in tone due to Botulinum toxin should be well established 6 weeks following injections. Cycles of 16 weeks have been chosen as any residual effect of treatment should be worn off by this time (19).

*Outcome measures:*

*Primary Outcome*

1. **Impact on caregiver burden**: Impact on caregiver burden in providing upper extremity care will be measured by the Carer Burden Scale (22, 23). The Carer Burden Scale consists of four items (cleaning the palm, cutting the fingernails, dressing, cleaning under the armpit). Each item will be rated by the study nurse (blinded to treatment allocation) on a five point Likert scale (“no difficulty” to “cannot do task”) at each time point (baseline, weeks 2, 6, 12, and 16 following injections) for a period of 2 days and scores will be averaged. Items scored will be summed and divided by the number of items answered giving a summary carer-burden score (0 = no carer burden🡪 4 = maximum carer burden).

**DEVIATION OF FINAL PROTOCOL FROM PROTOCOL SUBMITTED TO REB**

***As cutting fingernails was not consistently part of daily morning care in this population, it was omitted from the assessments. Since we included both left and right limbs, cleaning under the armpit and cleaning under the palm was scored for each side. As such, the total achievable score was 20. This was an oversite in the original protocol and became clear that scoring modification was required once study began.***

*Secondary Outcomes*

1. **Tone:** Tone will be assessed by the study co-ordinator using the Modified Ashworth Scale (MAS) of increased tone (7, 20). This is a 6 point scale grading the level of resistance encountered during manual passive stretching with 0=No increase in muscle tone, 1=slight increase in muscle tone, manifested by a catch and release, or by minimal resistance at the end of the range of motion when the affected part(s) is moved in flexion or extension, 2=Slight increase in muscle tone, manifested by a catch, followed by minimal resistance throughout the remainder (less than half) of the range of motion, 3=More marked increase in muscle tone through most of ROM, but affected part(s) easily moved, 4=Considerable increase in muscle tone, passive movement difficult, 5=Affected parts rigid in flexion and extension. Tone of the fingers, elbow, wrist, and shoulder abduction (depending on the muscles affected and injected) at baseline and after active and sham treatments, will be compared. A clinically significant response to treatment will be defined as at least a one point improvement from baseline in scores for muscle tone in the wrist and fingers (21).

**DEVIATION OF FINAL MANUSCRIPT FROM PROTOCOL SUBMITTED TO REB**

***While the MAS of increased tone was collected in study subjects, this information was not included in the final manuscript as it became clear during assessments that MAS was not a valid measure in paratonia due to its fluctuating nature (Waardenberg H, Elvers W, Van Vechgel F, Oostendorp R. Can paratonia be measured reliably? Evaluation of the reliability of a visual analogue scale and the modified tonus sclae of Ashworth for measuring paratonia. Nederlands Tijdschrift voor Fysiotherapie 1999;102:30-35).***

1. **Time to Perform Care:** Time to perform hygiene and dressing will be determined with video recording analysis prior to and following injections. Specifically, time in minutes to perform individual goals (dressing (upper body), cleaning under arm, cleaning palm, cutting nail, all tasks involved in the Carer Burden Scale) will be recorded by study nurse observing the professional caregiver and averaged over 2 days during assessments.

**DEVIATION OF FINAL MANUSCRIPT FROM PROTOCOL SUBMITTED TO REB**

***This data was collected but it became clear that this method of determining time it takes to accomplish morning routine was not feasible. There were different people performing morning care at each assessment in different ways and with variable efficiency despite trying to ensure prior to emrolment that this was uniform. For “time to perform morning care” to become a valid measure it would be necessary to standardize the protocol for administering care and by the same individual. As this study was a pilot study also looking at feasibility, we recognized that the way we collected this did not provide meaningful and valid data and chose to omit from our reporting. A future study on a larger scale (if performed) would need to consider these factors and record the time variable in a different way.***

1. **Visual Analogue Scale:** A caregiver burden visual analogue scale (VAS) will be completed by the professional caregiver caring for the patient in order to assess perception of ease of caregiving (16). A VAS will be used because it has been found to be valid in capturing subjective perceptions of health-related quality of life and other qualitative health outcomes (24). The VAS consists of a 100 mm line with anchors of 0 “giving care to the residents is very difficult” and 100 “giving care to the residents is very easy”.
2. **Global Assessment**: Overall response to treatment will be evaluated by the study investigator and by the professional caregiver caring for the patient using the global assessment scale (26) at each follow-up visit. A score of -4 indicates very marked worsening, 0 no change, and +4 very marked improvement.
3. **Pain:** As patients with advanced dementia are not able to express their experience of pain, a surrogate measure, the Pain Assessment in Advanced Dementia (PAINAD) Scale (25) will be used by the study nurse at each assessment time point to objectively evaluate correlates of pain and determine whether treatment with Botulinum toxin reduces the discomfort experienced during morning care. The PAINAD scale consists of 5 items (breathing, negative vocalization, facial expression, body language, and consolability) scored on a 0-2 point scale and then summed to arrive at a total score. The score will be averaged over 2 days

*Qualitative Assessments*

In addition to quantitative objective measures to determine reduction in burden, the professional caregiver will be questioned using open-ended interview techniques regarding what they have noted to be the most relevant impact from the intervention. They will be asked to provide any additional observations or comments that may not have been directly addressed by the study outcomes. This will provide insights into possible future research questions to be addressed and may provide possible other relevant outcome measures. The interviews will be carried out at 6 and 22 weeks by the clinical co-ordinator trained in qualitative research methods.

*Videos*

To document visually the impact of injections on postures and skin integrity, all subjects will be videotaped receiving morning care before injections and at each assessment following injections.

*Side Effects*

At each assessment, the professional caregiver will be asked about any adverse-effects they may have noticed as a consequence of the injections. They will be specifically questioned regarding excessive weakness, local skin effects including bruising or hypersensitivity, and distal spread effects such as breathing or swallowing impairment differing from before treatment. Any side-effect attributed to treatment will be monitored and recorded throughout the treatment period. If any serious adverse effects emerge (ie breathing or swallowing difficulty), treatment will be unblinded and appropriate interventions will be effected.

**Data collection and management**

SAS© computer software will be used to create a database. FSEDIT (SAS) will be used to maintain the database. Responses will be entered into the database as they become available using double data entry to minimize key stroke errors. Data cleaning will occur on an ongoing basis.

**Statistical Analysis**

*Power calculations*


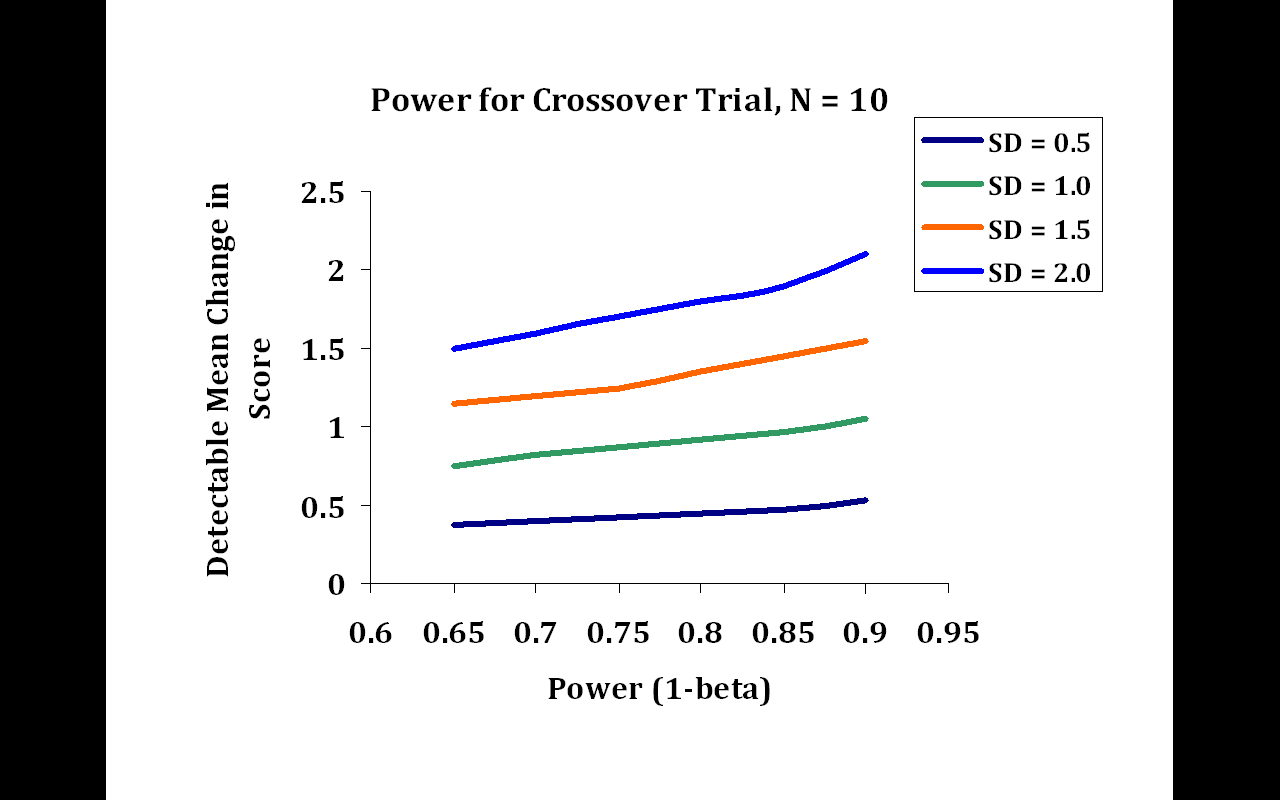
Power calculations performed using the xsampsi application for sample size and power in crossover trials (Stata v. 11.0) were used to create the figure below. Assuming 10 unique individuals are recruited into the study, we will have 70-80% power to detect mean changes of between 0.5 and 1.5 points on a 5 point Likert scale, under different assumptions regarding the standard deviation in such measurement (SD 0.5, 1, 1.5 or 2). As such, we expect to be able to detect clinically meaningful changes in measurement with treatment even with the relatively small sample size anticipated in this pilot study. Data collection will permit further refinement of power and sample size calculations for future, larger trials if effects observed here are promising.

*Statistical Analysis*

Because of between patient variability in the affected area injected and severity of paratonia, and because of our desire to control for period and ordering effects we will use mixed regression models for analyses of quantitative outcomes, in order to isolate the effect of Botulinum toxin relative to placebo. Mixed models as applied to crossover designs permit adjustment for treatment sequences (i.e., Botulinum toxin first vs. second), period effects (i.e., first injection vs. second injection), between individual variation in severity and response to therapy, etc. We will estimate treatment fixed effects (i.e., effect of Botulinum toxin treatment), defined as the change in relevant scores from baseline to 6 weeks, and 16 to 22 weeks (i.e., change from baseline until 6 weeks post-treatment or placebo). Because of the anticipated variability in severity of paratonia in individual patients, and variability in responses of caregivers to patient disability, we will treat individual patient/caregiver dyads as model random effects.

**Study team**

An interdisciplinary team with expertise in quality of life, dementia research and Botulinum toxin administration has been assembled.

*Principal Investigator:* **Dr. Galit Kleiner-Fisman MD, FRCPC**, an Assistant Professor of Neurology at the University of Toronto, is a specialist in Movement Disorders and spasticity management. Her clinical practice is focused on movement disorders in geriatric patients. She has experience in clinical outcomes research and has been administering Botulinum toxin to patients with movement disorders and spasticity for 9 years. She will be responsible for the study overall as well as recruitment of study patients, administration of injections, analysis and reporting of data.

*Collaborators:* **Dr. Mario Masselis, MD, MSc, FRCPC and Dr. Tiffany Chow** are both internationally recognized behavioural neurologists working out of Sunnybrook Hospital and Baycrest Hospital respectively with large dementia populations in their memory Clinics. They will be responsible for selecting and referring appropriate candidates for the study and will be involved in data reporting.

**DEVIATION OF FINAL MANUSCRIPT FROM PROTOCOL SUBMITTED TO REB**

***Drs. Masselis and Chow did not refer patients from their Clinics and did not participate in the study and so were not included in the manuscript***

*Methodological consultant*: **Dr. Gary Naglie** **MD, FRCPC**: Dr. Naglie is a geriatrician, a senior scientist, and the Mary Trimmer Chair in Geriatric Medicine Research at University of Toronto. He is leading a CIHR-funded researcher on quality of life in AD and has contributed fundamentally to study design. He will oversee data analysis and reporting to ensure methodological rigor and accuracy.

**DEVIATION OF FINAL MANUSCRIPT FROM PROTOCOL SUBMITTED TO REB**

***Dr. Naglie did not participate in the study and so was not included in the manuscript as he did not meet authorship criteria. He was involved in initial discussions about the protocol and for this was included as an author for the abstract (please see “Knowledge translation for citation of published abstract).***

*Clinical co-ordinator (TBA)*

The clinical co-ordinator will be responsible for ensuring the administration for the overall conduct of the study including scheduling appropriate patients once they have been screened, assisting in REB submission and compliance, collecting outcome measures, entering data and overseeing completeness of data. Following training by the Elkie Adler Clinic’s physical therapist, the clinical co-ordinator will provide support and re-enforcement to caregivers as to the specific exercise regimens to be applied to patients.

**Feasibility**

The Elkie Adler Clinic has over 4,000 patient visits per yr, and serves patients with neurological impairments including dementia, multiple sclerosis, stroke, traumatic brain injury, Parkinson’s disease, and spinal cord injuries. Though it serves a broad population of outpatients distributed throughout the GTA and Ontario, it increasingly treats clients who reside in long-term care institutions, who constitute natural subjects for this study as they are referred to the clinic primarily for the indication that this study proposes to investigate. Both Dr. Chow and Dr. Masselis have a large population of patients with cognitive impairments that would be eligible for the study (please refer to letters of support). As such, recruitment of 1-2 patients/month is a feasible target with recruitment completed within 6-12 months.

**Ethical considerations**

The protocol will be approved by the Baycrest Research Ethics Board. Study forms and the computerized study database will contain no personal identifiers. All paper forms will be kept in locked cabinets and all computerized files will be kept on password protected computers that will only be accessible to study personnel.

**Knowledge translation**

Study findings will be submitted for presentation at an international neurology meeting (e.g., American Academy of Neurology, American Neurological Association, Movement Disorders Society Congress, etc). Study findings will be submitted for publication in a high impact specialty or subspecialty journal, with preference given to open-access journals due to their enhanced availability to practicing clinicians.

***Study was presented at the 18^th^ International Movement Disorders Congress in Stockholm Sweden June 11, 2014 and the abstract has been published (****Kleiner-Fisman, G., Khoo, E., Moncrieffe, N., Forbell, T., Gryfe, P., Naglie, G., Fisman, D.; Botulinum toxin treatment reduces involuntary postures and caregiver burden in people with advanced cognitive impairment [abstract]. Movement Disorders 2014;29 Suppl 1 :1107)*****

**Potential Impact/Significance of Study**

In these times of fiscal constraint, difficult decisions need to be made regarding resource allocation to optimize patient care. If the potential for this intervention is affirmed through well designed clinical trials, this would constitute an important, attractive, and likely cost-effective management tool. Our expectation is that the use of Botulinum toxin injections for management of paratonia in individuals with dementia is an intervention with the capacity to reduce care burden (including reduced time to perform ADLs), improve standard of care for dependent individuals, and potentially increase patient quality of life in this increasingly common condition.

**BUDGET**

| **Personnel** | | | | | | |
| --- | --- | --- | --- | --- | --- | --- |
| **Name** | **Role** | **% Effort** | **Base salary** | **Fringe Benefits** | **Total salary** | **Total Requested** |
| G. Kleiner-Fisman | PI | 20% |  |  |  | In kind |
| TBA | Clinical co-ordinator |  | $50,000.00 | $11,000.00 | $61,000.00 |  |
| Year 1 |  | 50% |  |  |  | $30,500.00 |
| Year 2 |  | 50% |  |  |  | $30,500.00 |
| Mario Masselis | Collaborator | 5% |  |  |  | In kind |
| Tiffany Chow | Collaborator | 5% |  |  |  | In kind |
| Gary Naglie | Methodological consultant (MC) | 5% |  |  |  | In kind |
| Statistical support | Data analysis | $65/hr x 50 hrs | | | | $3250.00 |
| **Materials** | | | | | |  |
| Computer | | | | | | $2000.00 |
| Desk/cabinet for Research nurse | | | | | | $500.00 |
| Computer software (SAS license), network access ($2000/yr) | | | | | | $4000.00 |
| Storage of documents fee (10 yrs) | | | | | | $1500.00 |
| Office services (copying, printing) | | | | | | $300.00 |
| Botulinum toxin (~4 vials/subject) | | | | | | Provided |
| EMG needles (2/subject) | | | | | | $650.00 |
| Syringes, saline, alcohol, gauze | | | | | | $200.00 |
| Stop-watch | | | | | | $100.00 |
| Video camera | | | | | | $600.00 |
| **Pharmacy costs** | | | | | |  |
| Ordering, labeling, blinding, recording and dispensing of trial drug | | | | | | $1350.00 |
| **IRB and Scientific Review** (Baycrest Hospital) | | | | | | $2700.00 |
| **Travel** | | | | | |  |
| Meeting attendance (hotel x 4 days, airfare, meeting registration, food x 4 days) x 1 | | | | | | $2500.00 |
| Meeting presentation materials (poster) x 1 | | | | | | $200.00 |
| **TOTAL direct costs** | | | | | | **$80,850.00** |
| **Indirect costs (10%)** | | | | | | **$8085.00** |
| **TOTAL** | | | | | | **$88,935.00** |

Reference List

(1) Smetanin P, Kobak P, Briante C, Ahmad S. *Rising Tide: The Impact of Dementia in Canada 2008 to 2038*. RiskAnalytica; 2009.

(2) Canadian study of health and aging: study methods and prevalence of dementia. *CMAJ* 1994 Mar 15;150:899-913.

(3) Matthews FE, Dening T. Prevalence of dementia in institutional care. *Lancet* 2002 Jul 20;360:225-226.

(4) Fox PJ, Kohatsu N, Max W, Arnsberger P. Estimating the costs of caring for people with Alzheimer disease in California: 2000-2040. *J Public Health Policy* 2001;22:88-97.

(5) Knapp M, Comas-Herrera, Somani A, Banerjee S. Dementia:
Summary report for the National Audit Office
international comparisons. London: Personal Social Services Research Unit
London School of Economics and Political Science
and
Section of Mental Health and Ageing
The Institute of Psychiatry, King's College London; 2007.

(6) Dupre E. Debilite mentale and debilite motrice associees. *Rev Neurol* 1910;20:54-56.

(7) Hobbelen JS, Koopmans RT, Verhey FR, Habraken KM, de Bie RA. Diagnosing paratonia in the demented elderly: reliability and validity of the Paratonia Assessment Instrument (PAI). *Int Psychogeriatr* 2008 Aug;20:840-852.

(8) Franssen EH, Kluger A, Torossian CL, Reisberg B. The neurologic syndrome of severe Alzheimer's disease. Relationship to functional decline. *Arch Neurol* 1993 Oct;50:1029-1039.

(9) Hobbelen JS, Verhey FR, Bor JH, de Bie RA, Koopmans RT. Passive movement therapy in patients with moderate to severe paratonia; study protocol of a randomised clinical trial (ISRCTN43069940). *BMC Geriatr* 2007;7:30.

(10) Souren LE, Franssen EH, Reisberg B. Neuromotor changes in Alzheimer's disease: implications for patient care. *J Geriatr Psychiatry Neurol* 1997 Jul;10:93-98.

(11) Kao I, Drachman DB, Price DL. Botulinum toxin: mechanism of presynaptic blockade. *Science* 1976 Sep 24;193:1256-1258.

(12) Brashear A, Gordon MF, Elovic E, et al. Intramuscular injection of botulinum toxin for the treatment of wrist and finger spasticity after a stroke. *N Engl J Med* 2002 Aug 8;347:395-400.

(13) Elovic EP, Brashear A, Kaelin D, et al. Repeated treatments with botulinum toxin type a produce sustained decreases in the limitations associated with focal upper-limb poststroke spasticity for caregivers and patients. *Arch Phys Med Rehabil* 2008 May;89:799-806.

(14) Giovannelli M, Borriello G, Castri P, Prosperini L, Pozzilli C. Early physiotherapy after injection of botulinum toxin increases the beneficial effects on spasticity in patients with multiple sclerosis. *Clin Rehabil* 2007 Apr;21:331-337.

(15) Simpson DM, Gracies JM, Yablon SA, Barbano R, Brashear A. Botulinum neurotoxin versus tizanidine in upper limb spasticity: a placebo-controlled study. *J Neurol Neurosurg Psychiatry* 2009 Apr;80:380-385.

(16) Wells DL, Dawson P, Sidani S, Craig D, Pringle D. Effects of an abilities-focused program of morning care on residents who have dementia and on caregivers. *J Am Geriatr Soc* 2000 Apr;48:442-449.

(17) Kanovsky P, Slawek J, Denes Z, et al. Efficacy and safety of botulinum neurotoxin NT 201 in poststroke upper limb spasticity. *Clin Neuropharmacol* 2009 Sep;32:259-265.

(18) Delagi EF, Perotto A, Iazzetti J, Morrison D. Anatomic Guides for the Elecromyographer. In: Charles C.Thomas, ed., 2nd ed Springfield, 1980.

(19) Brin MF. Dosing, administration, and a treatment algorithm for use of botulinum toxin A for adult-onset spasticity. Spasticity Study Group. *Muscle Nerve Suppl* 1997;6:S208-S220.

(20) Waardenburg H, Elvers W, Van Vechgel F, Oostendorp R. Can paratonia be measured reliably? Evaluation of the reliability of a visual analogue scale and the modified tonus sclae of Ashworth for measuring paratonia. *Nederlands Tijdschrift voor Fysiotherapie (in dutch)* 1999;102:30-35.

(21) Albright AL, Barron WB, Fasick MP, Polinko P, Janosky J. Continuous intrathecal baclofen infusion for spasticity of cerebral origin. *JAMA* 1993 Nov 24;270:2475-2477.

(22) Bhakta BB, Tennant A, Cozens JA, et al. Application of item response theory to measure the disabling effects of severe upper limb spasticity in stroke and the consequent carer burden. *Cerebrovascular Dis* 1999;9:124.

(23) Bhakta BB, Cozens JA, Chamberlain MA, Bamford JM. Impact of botulinum toxin type A on disability and carer burden due to arm spasticity after stroke: a randomised double blind placebo controlled trial. *J Neurol Neurosurg Psychiatry* 2000 Aug;69:217-221.

(24) Waltz CFSOL, Lenz ER. Measurement in Nursing Research, 2nd ed. Philadelphia: F.A. Davis, 1991.

(25) Warden V, Hurley AC, Volicer L. Development and psychometric evaluation of the Pain Assessment in Advanced Dementia (PAINAD) scale. *J Am Med Dir Assoc* 2003 Jan;4:9-15.

(26) Naumann M, Yakovleff A, Durif F. A randomized, double-masked, crossover comparison of the efficacy and safety of botulinum toxin type A produced from the original bulk toxin source and current bulk toxin source for the treatment of cervical dystonia. *J Neurol* 2002 Jan;249:57-63.
